# Supplementary material for: Extracellular Vesicles as Drug Carriers for Enzyme Replacement Therapy to Treat CLN2 Batten Disease: Optimization of Drug Administration Routes
Source: Cells. 2020 May 20;9(5):1273. doi: 10.3390/cells9051273 (PMC7290714; doi:10.3390/cells9051273)
Supplement: Supplementary file 1 [file cells-09-01273-s001.pdf]

Supplementary Materials

Extracellular Vesicles as Drug Carriers for Enzyme Replacement Therapy to Treat CLN2 Batten Disease: Optimization of Drug Administration Routes

Matthew J. Haney [1,2], Yuling Zhao [1,2], Yeon S. Jin [2] and Elena V. Batrakova [1,2,*]

[1] Center for Nanotechnology in Drug Delivery, University of North Carolina at Chapel Hill, Chapel Hill, NC 27599, USA; mjhaney@email.unc.edu (M.J.H.); yulingz@email.unc.edu (Y.Z.)
[2] Eshelman School of Pharmacy, University of North Carolina at Chapel Hill, Chapel Hill, NC 27599, USA; ysjin@email.unc.edu
* Correspondence: batrakov@ad.unc.edu; Tel.: 919-537-3712

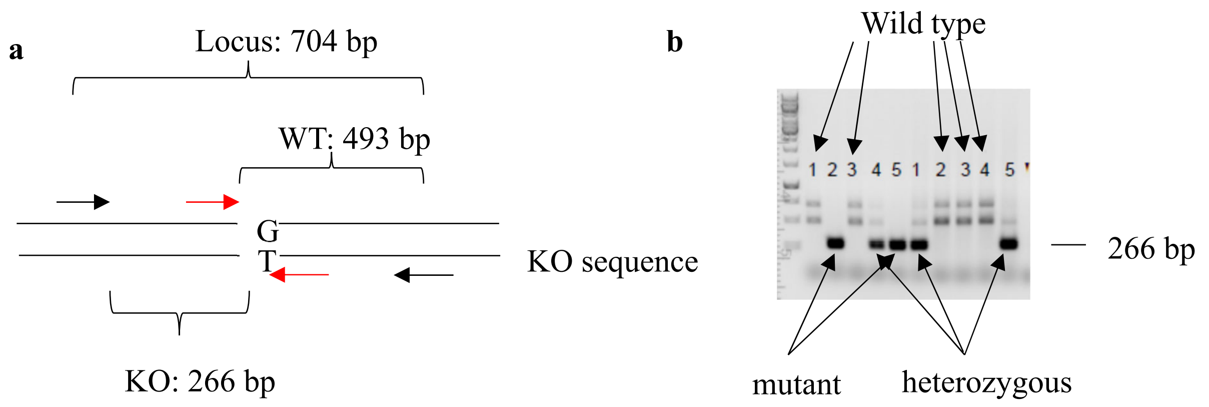

Supplementary Figure S1. Batten disease model, LINCL mice. Pups of knock-out CLN2 KO mice were subjected to PCR analysis to identify mutant animals (#2 and #5) that were utilized then for EVs biodistribution studies. (**a**): structure of CLN2 gene, (**b**): detection of CLN2 mRNAs in mutant and control mice.

a

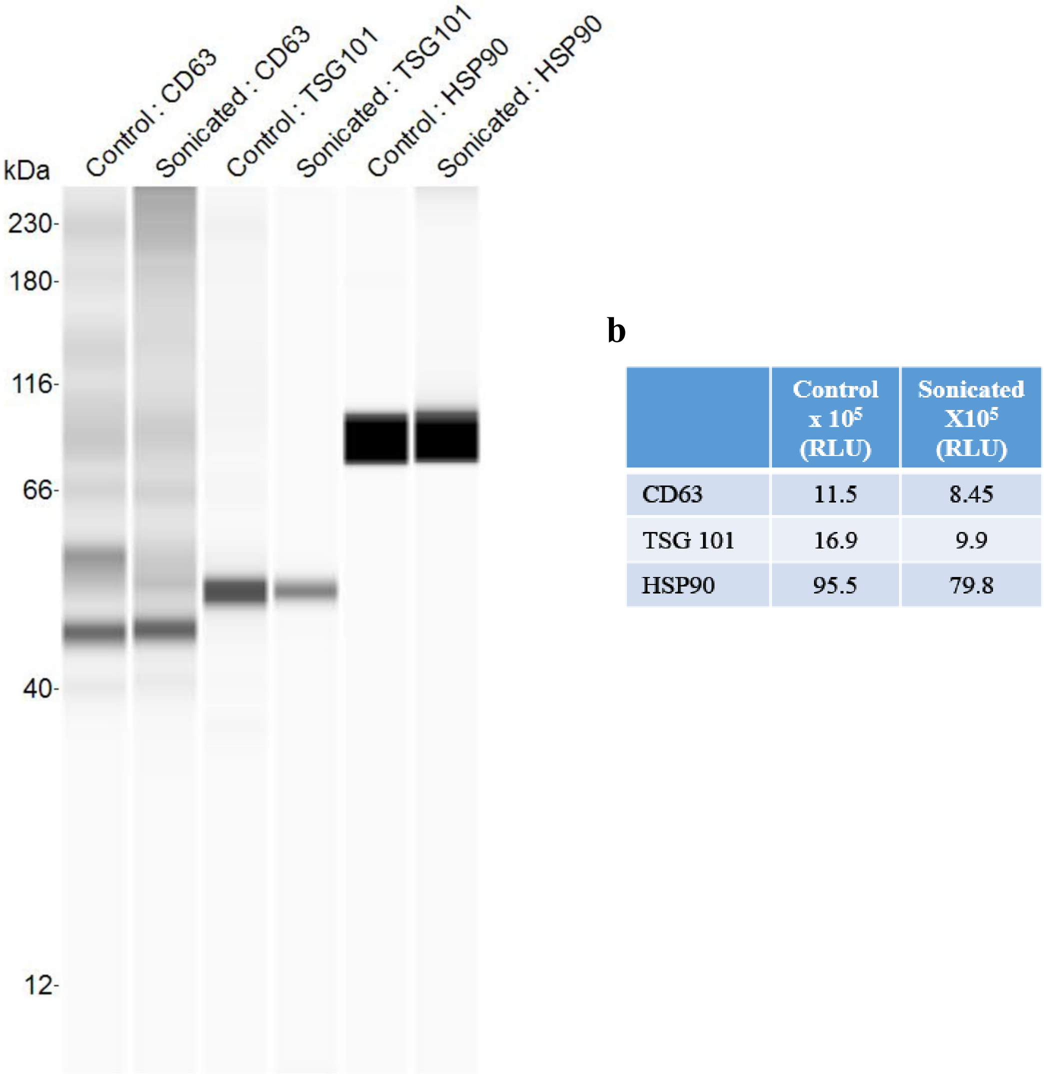

b

	Control x 10^5 (RLU)	Sonicated X10^5 (RLU)
CD63	11.5	8.45
TSG 101	16.9	9.9
HSP90	95.5	79.8

Supplementary Figure S2. Characterization of EV nanocarriers by Western blot analysis. Macrophage-derived EVs were subjected sonication as described in Materials and Methods section, purified through the column, and the levels of EV-specific proteins (CD63, TSG101, and HSP 90) were visualized (**a**), and quantified (**b**) using West. Followed by sonication procedure, EVs retained specific markers, although their levels were slightly decreased.

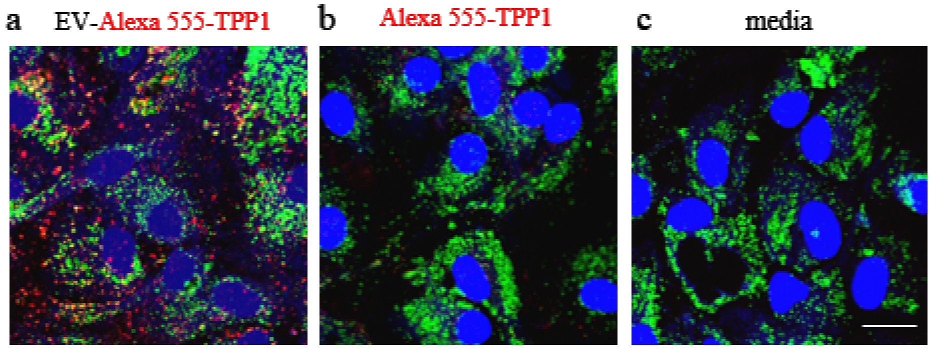

Supplementary Figure S3. Accumulation of fluorescently labeled EV-TPP1 in primary neurons *in vitro*. Primary neurons were supplemented with (**a**) fluorescently labeled Alexa 555-TPP1 loaded in EVs (10^{11} particles/mL), or (**b**) Alexa 555-TPP1 alone, or (**c**) TPP1-free media for 4h. Then, cells were washed with ice-cold PBS, lysosomes were stained with Ab to LAMP1 over night, and mounted with nuclei staining DAPI (blue). EVs facilitated enzyme transport resulting in higher Alexa 555 signal accumulation in primary neurons treated with EV-TPP1 (**a**) compared with TPP1 alone (**b**). The bar: 20 μm.

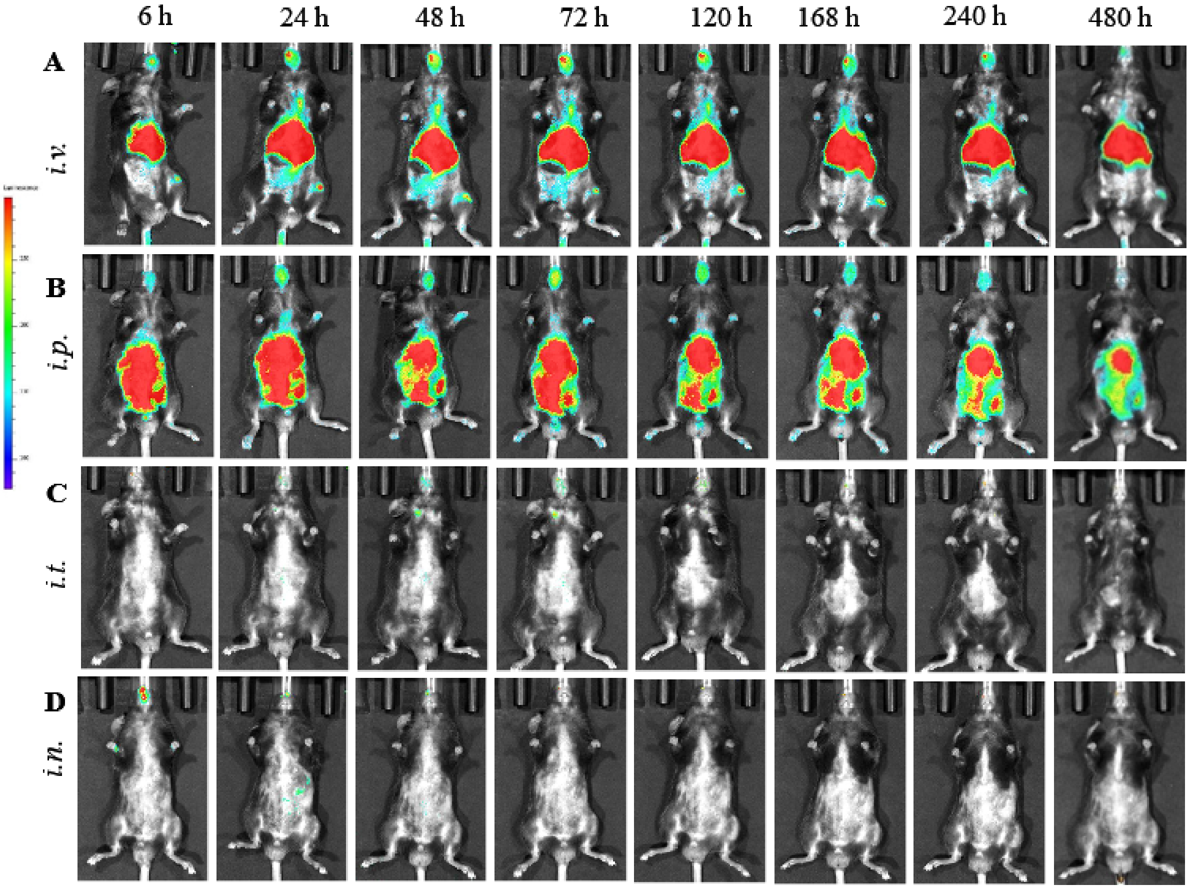

Supplementary Figure S4. Biodistribution of DIR-EVs in CLN2 KO mice by IVIS. Labeled with fluorescent hydrophobic dye, DIR- EVs were injected into BD mice (1 mo. of age, $3x10^{12}$ particles/mL) though (**A**): *i.v.* (200 μL), (**B**): *i.p.* (200 μL), (**C**): *i.t.* (50 μL), or (**D**): *i.n.* (20 μL) routes, and imaged by IVIS up to 480 h. Supine representative images from same treatment groups as in Figure 1 show significant amount of DIR-EVs was accumulated in main excretion organs, liver, spleen, and kidney, especially for *i.v.* and *i.p.* administration routes.

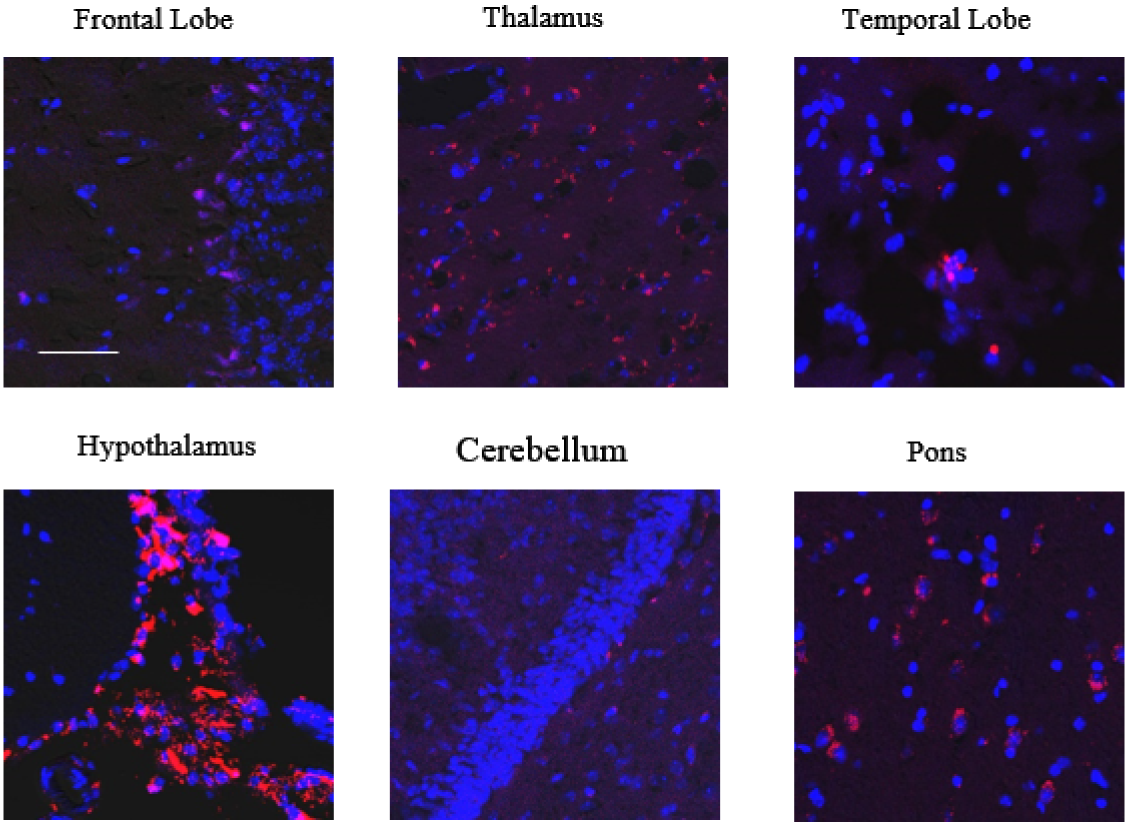

Supplementary Figure S5. Accumulation of fluorescently labeled EV-TPP1 formulations in different regions of CLN2 mouse brain. CLN2 mice were injected with non-labeled EVs loaded with fluorescently labeled Alexa 555-TPP1 (red) through *i.t.* administration rout. 48 hours later, mice were sacrificed, perfused, and brain slides were mounted on slides with nuclei DAPI staining (blue) and studied by confocal microscopy. Significant amount of TPP1 was delivered in EVs nanocarriers to all studies brain regions, especially hypothalamus, thalamus, and pons. The bar: 50 μm.

Supplementary Table S1. Therapeutic Effects of EV-TPP1 treatments in BD mice [a]

	CLN2 (RFU)					WT/saline
	saline	*i.v.*	*i.p.*	*i.t.*	*i.n.*	
Neuronal survival	60 ± 5 (#)	119 ± 6 (*)	139 ± 3 (*)	151 ± 21 (*)	135 ± 11 (*)	170 ± 34 (*)
Astrocytosis	411 ± 9 (#)	124 ± 11 (*)	130 ± 14 (*)	106 ± 5 (*)	89 ± 11 (*)	156 ± 30 (*)
Lysosomal storage	252 ± 12 (#)	198 ± 33	183 ± 34	101 ± 32 (*)	92 ± 28 (*)	106 ± 14 (*)

[a] CLN2 KO mice were treated with EV-TPP1 formulations through different routes as in Figure 4. BD mice and WT littermates injected with saline were used in control groups. Three weeks later, mice were sacrificed, perfused, brain was harvested, and slide were stained with Ab to neurons (NeuN), Ab to activated astrocytes (GFAP), or lysosomal storage bodies (subunit c of mitochondrial ATP synthase). The slides were examined by confocal microscopy, and the quantification of mean fluorescence signals was assessed.

[b] Statistical significance is shown by asterisk: $*p < 0.05$ compared to *Cln2* mice with saline injections, or $\#p < 0.05$, compared to WT littermates with saline injections (healthy controls). $N = 7$.
